# Analyzing the field of bioinformatics with the multi-faceted topic modeling technique

**DOI:** 10.1186/s12859-017-1640-x

**Published:** 2017-05-31

**Authors:** Go Eun Heo, Keun Young Kang, Min Song, Jeong-Hoon Lee

**Affiliations:** 1 Department of Library and Information Science, Yonsei University, 50 Yonsei-ro Seodaemun-gu, Seoul, 03722 Republic of Korea; 2 Department of Creative IT Engineering, POSTECH, 77 Cheongam-ro Nam-gu, Pohang, Gyeongbuk 37673 Republic of Korea

**Keywords:** Bioinformatics, Text mining, Topic modeling, ACT model, Keyphrase extraction

## Abstract

**Background:**

Bioinformatics is an interdisciplinary field at the intersection of molecular biology and computing technology. To characterize the field as convergent domain, researchers have used bibliometrics, augmented with text-mining techniques for content analysis. In previous studies, Latent Dirichlet Allocation (LDA) was the most representative topic modeling technique for identifying topic structure of subject areas. However, as opposed to revealing the topic structure in relation to metadata such as authors, publication date, and journals, LDA only displays the simple topic structure.

**Methods:**

In this paper, we adopt the Tang et al.’s Author-Conference-Topic (ACT) model to study the field of bioinformatics from the perspective of keyphrases, authors, and journals. The ACT model is capable of incorporating the paper, author, and conference into the topic distribution simultaneously. To obtain more meaningful results, we use journals and keyphrases instead of conferences and bag-of-words.. For analysis, we use PubMed to collected forty-six bioinformatics journals from the MEDLINE database. We conducted time series topic analysis over four periods from 1996 to 2015 to further examine the interdisciplinary nature of bioinformatics.

**Results:**

We analyze the ACT Model results in each period. Additionally, for further integrated analysis, we conduct a time series analysis among the top-ranked keyphrases, journals, and authors according to their frequency. We also examine the patterns in the top journals by simultaneously identifying the topical probability in each period, as well as the top authors and keyphrases. The results indicate that in recent years diversified topics have become more prevalent and convergent topics have become more clearly represented.

**Conclusion:**

The results of our analysis implies that overtime the field of bioinformatics becomes more interdisciplinary where there is a steady increase in peripheral fields such as conceptual, mathematical, and system biology. These results are confirmed by integrated analysis of topic distribution as well as top ranked keyphrases, authors, and journals.

**Electronic supplementary material:**

The online version of this article (doi:10.1186/s12859-017-1640-x) contains supplementary material, which is available to authorized users.

## Background

Over the years, academic subject areas have converged to form a variety of new, interdisciplinary fields. Bioinformatics is one example. Research domains from molecular biology to machine learning are used in conjunction to better understand complex biological systems such as cells, tissues, and the human body. Due to the complexity and broadness of the field, bibliometric analysis is often adopted to assess the current knowledge structure of a subject area, specify the current research themes, and identify the core literature of that area [1].

Bibliometrics identifies research trends using quantitative measures such as a researcher’s number of publications and citations, journal impact factors, and other indices that can measure impact or productivity of author or journal [2–5]. In addition, other factors such as the affiliation of authors, collaborations, and citation data are often incorporated into bibliometric analysis [6–9].

Previous studies mainly rely on quantitative measures and suffer from the lack of content analysis. To incorporate content analysis into bibliometrics, text-mining techniques are applied. Topic-modeling techniques are mostly adopted to identify the topics of a subject area while analyzing that area more abundantly [10–13]. These techniques allow for enriched content analysis. As an extension of Latent Dirichlet Allocation (LDA), which is the best received topic-modeling technique, Steyvers et al. [14] proposed the author-topic modeling technique that analyzes authors and topics simultaneously. They identify the authors’ impact or productivity of researchers in a given subject area [15, 16]. By adding multiple conditions to LDA, Tang et al. [17] suggested a new methodology, called the Author-Conference-Topic (ACT) model that analyzes the author, conference, and topic in one model to understand the subject area in an integrated manner.

In this paper, we apply the ACT model to examine interdisciplinary nature of bioinformatics. Unlike studies that use extended versions of LDA for topic analysis, the ACT model enables us to analyze topic, author, and journal at one time, providing an integrated view for understanding bioinformatics. The research questions that we are to investigate in this paper are: 1) What are the topical trends of bioinformatics over time? 2) Who are the key contributors in major topics of bioinformatics?, and 3) Which journal is leading which topic?

To address these questions, we collect PubMed articles in XML format and extract metadata and content such as the PMID, author, year, journal, title, and abstract. From the title and abstract, we extract keyphrases, which provide more meaningful interpretations than single words, as an input of the ACT model. We also divide the collected datasets into four time periods to examine the topic changes over time. The results of ACT model–based analysis show that various topics begin to appear and mixed subject topics become more apparent over time.

The rest of the paper is organized as follows. In the Background section, we discuss work related to bibliometric analysis and topic modeling. We then describe the proposed method in the Methods section. We analyze and discuss the results of leading topics, authors, and journals in the Result and Discussion section. Finally, we conclude the paper and suggest future lines of inquiry in Conclusions.

## Related work

### Bibilometric analysis

Bibliometric analysis identifies the research trends in a given subject area and core journals or documents, and helps with contrastive analysis. Many bibliometric studies use the number of published articles or journal impact factors to measure research productivity or to identify core journals in a specific field. Soteriades and Falalgas [3] applied quantitative and qualitative measurements to analyze the fields of preventive medicine, occupational and environmental medicine, epidemiology and public health using the number of articles and impact factor. Ugolini et al. [4] measured research productivity and evaluated the publication trends in the field of cancer molecular epidemiology. To quantify productivity, they used the number of articles and average and sum of impact factors. To evaluate publication trends, they collected and divided the keywords from MeSH terms about the publication into six groups. Ramos et al. [18] measured the national research activity of the tuberculosis field, using impact factor and the first author’s address. Claude et al. [19] examined research productivity by using distribution of publications related to medicine and ANN, the subfield of biology. They used the number of publications, impact factor, and journal category compared with national gross domestic product (GDP). In the bioinformatics field, Patra and Mishra [20] used the number of articles, publication of each journal, publication type, and the impact factor of journals to understand the growth of bioinformatics. They also found the core journals in the bioinformatics fields. Using author affiliation, they applied Lotka’s law to assess the distribution of each author’s productivity. Chen et al. [2] identified research trends using statistical methods based on the type of publication, language, and distribution of nation or institution. They measured h-index, adding statistical materials with the number of citations. Through this, they analyzed the research productivity by topic, institution, and journal. In addition, they conducted a keyword analysis to comprehend the research trend in a macroscopic view.

Mainstream bibliometrics research focuses on identifying the knowledge structure of a certain field with quantitative measures. In addition, some studies use author information or the collaboration pattern among authors to understand the certain field. Seglen and Aksnes [9] used the size and the productivity of research groups in the microbiology field in Norway as a measurement for bibliometric analysis. Geaney et al. [7] performed bibliometric analysis and density-equalizing mapping on scientific publications related to type 2 diabetes mellitus. They collected citation data and used various citation-oriented measures such as the number of citations, the average number of citations per journal, the total number of publications, impact factor, and eigenfactor score. To conduct content analysis and study the collaboration pattern between authors and the core sub-field of AIDS, Macías-Chapula and Mijangos-Nolasco [8] analyzed MeSH thesaurus using check tags, main headings, and subheadings of each MeSH term hierarchy. In addition, to measure the national research productivity, they used the authors’ address information. Bornmann and Mutz [6] recently identified the development of modern science by bibliometric analysis. They divide the data into three time periods to analyze the changes of fields over time.

### Text mining applied to bibliometrics

Recently, there have been many attempts to apply text-mining techniques to bibliometric analysis to identify the knowledge structure of the field or measure its influence on other researchers and their fields and productivity. Song and Kim [11] collected full-text articles from PubMed Central and computed their citation relation. They infer the knowledge structure and understand the trend of the bioinformatics field. In a similar vein, Song et al. [12] measured the influence and productivity of bioinformatics by mining full-text articles retrieved from PubMed Central. To calculate the field’s productivity, they identified the most productive author, nation, institution, and topic word; to calculate its influence, they identified the most-cited paper, author, and rising researcher. Song et al. [21] analyzed topic evolution in the bioinformatics field using DBLP data in the field of Computer Science. To identify topic trends over time, they divided a dozen years (2000–2011) into four periods and applied the Markov Random Field-based topic clustering. For automatic clustering labeling, they calculated topic similarity based on Within-Period Cluster Similarity (WPCS) and Between-Period Cluster Similarity (BPCS). Their approach created topic graphs that show interaction among topics over someperiod of time. Lee et al. [22] mapped the Alzheimer’s disease field in three different perspectives: indexer, author, and citer. They applied entity-metrics [23] the extended notion of bibliometrics, to analyze the field by constructing four kinds of networks that convey these three perspectives.

These studies identify the knowledge structure of a certain field by constructing bibliometric networks or databases with text-mining techniques. The most prevalent approach is to apply topic modeling to content analysis as a part of bibliometrics. Starting from the probabilistic Latent Semantic Indexing (pLSI) [24] model, Latent Dirichlet Allocation (LDA) [25] is the most accepted topic modeling technique for bibliometrics. While each document consists of a set of topics in pLSI, using the LDA model a more precise manipulation is added to organize the topics. Yan [13] used the LDA model to measure the influence and popularity of library and information science. He also identified the most-cited area and the patterns in this field. Jeong and Song [10]’s research measured the time gap among three different resources—web, patent, and scientific publication—in two research domains by applying the LDA model. The basic input unit for LDA is a set of documents. To organize author information into topics, Rosen-Zvi et al. [16] and Steyvers et al. [14] proposed the author-topic model with different theoretical background. Li et al. [15] identified the relations between authors and topics by using the author-topic model. They analyze the topic distribution to examine how many authors are associated with a certain topic. Also through the number of authors, they identify topics that are studied by many researchers. Tang et al. [17] proposed the ACT model which identifies paper, author, and conference simultaneously. Additionally, they developed the ArnetMiner system for mining academic research social networks.Tang et al. [26] also supplement ArnetMiner for a topic level expertise search over heterogeneous networks using the ACT model. It generates the most issued topics, author’s interestedness, paper search, academic suggestion, and experts in a specific field. Kim et al. [27] adopted the ACT model in terms of citation analysis. They collected the dataset in the field of oncology from PubMed Central, which provides the full-text articles in the biomedical field. They utilized the ACT model for analyzing citation sentences and journals instead of abstracts and conferences.

In conclusion, most previous studies identified knowledge structures by adopting not only bibliometric analysis but text-mining techniques such as the LDA model. To supplement bibliometric analysis, there are many attempts to incorporate content analysis into bibliometrics by adopting the LDA model text-mining techniques. However, the main limitation of this application of the LDA model,the representative method for trend analysis, is that it only explains topical trends by using one parameter such as bag-of-words on documents via topical terms. It is not sufficient to conduct comprehensive analysis for understanding knowledge disciplines. Therefore, in this paper, we apply the ACT model to the bioinformatics field for integrated analysis. Applying the ACT model, we aim to explore the importance of authors and journals in relation to topics. We divided the collected datasets into four periods to trace the changes of topic, author, journal ranking over time, and combine the results with bibliometric analysis.

## Methods

**Fig. 1:**
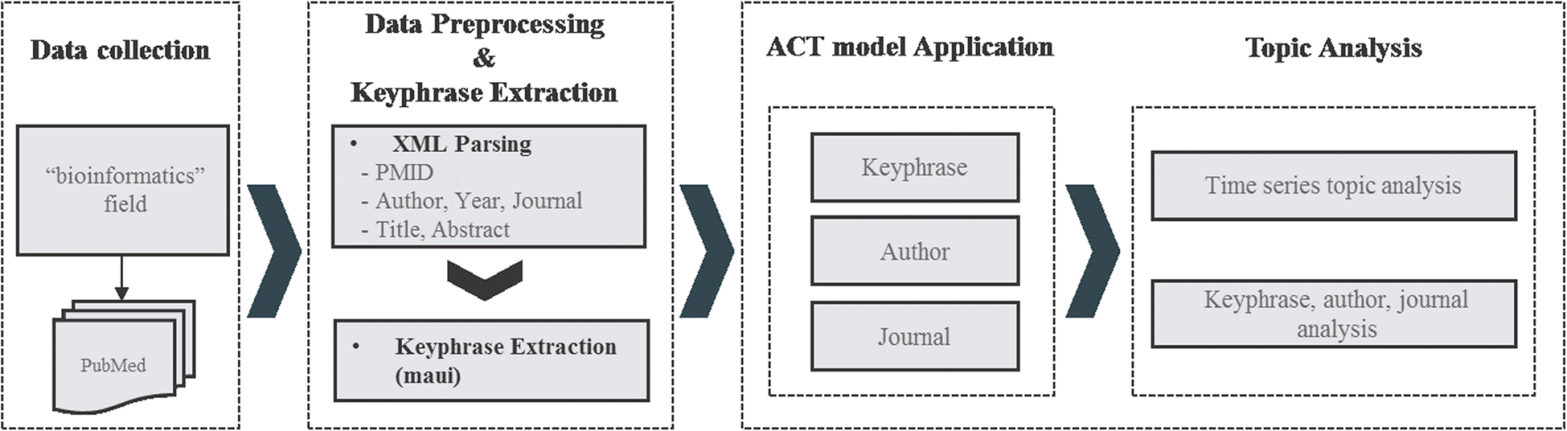
Research overflow. Research overflow of our approach consists of data collection, preprocessing, keyphrase extraction, ACT model application, and topic analysis

In this section, we describe data collection, preprocessing, and keyphrase extraction to feed input into the ACT model. Figure 1 illustrates the overflow of our approach; detailed descriptions of each component are provided in the following section.

### Data collection

For analysis, we collect 48 journals belonging to the bioinformatics field used by Song and Kim [11]. Forty-six out of the 48 journals are found via the advanced search tool provided by PubMed. Two journals, Advanced Bioinformatics and Genome Integration, are not retrieved from PubMed. We download the 46 PubMed-listed journals in XML format (Table 1). The total number of papers indexed in these journals is 241,569; Biochemistry had the greatest number of papers with 62,270, accounting for 25.78% of the collected publications.

**Table 1:** Statistics of collected publications

Ranking	Journal Name	Number of Papers	Ratio (%)
1	Biochemistry	62,270	25.78
2	Journal of Molecular Biology	29,968	12.41
3	The EMBO Journal	17,296	7.16
4	Journal of Theoretical Biology	12,200	5.05
5	Bioinformatics	9,847	4.08
6	Human Molecular Genetics	9,347	3.87
7	Genomics	8,316	3.44
8	BMC Genomics	7,741	3.20
9	BMC Bioinformatics	6,780	2.81
10	Protein Science : a publication of the Protein Society	6,047	2.50
11	Journal of Proteome Research	5,575	2.31
12	Proteomics	5,545	2.30
13	Journal of Biotechnology	5,204	2.15
14	PLOS Genetics	5,139	2.13
15	PLOS Computational Biology	3,852	1.59
16	BMC Research Notes	3,743	1.55
17	Mammalian Genome	3,499	1.45
18	Genome Biology	3,411	1.41
19	PLOS Biology	3,280	1.36
20	Trends in Biochemical Sciences	3,171	1.31
21	Trends in Genetics	3,035	1.26
22	Journal of Molecular Modeling	2,852	1.18
23	Molecular & cellular proteomics : MCP	2,796	1.16
24	Trends in Biotechnology	2,353	0.97
25	Bulletin of Mathematical Biology	2,331	0.96
26	Journal of Proteomics	2,158	0.89
27	Physiological Genomics	1,794	0.74
28	Journal of Computer-Aided Molecular Design	1,706	0.71
29	BMC Systems Biology	1,397	0.58
30	Bioinformation	1,297	0.54
31	Pharmacogenetics and Genomics	1,072	0.44
32	Statistical Methods in Medical Research	976	0.40
33	Journal of ComputationalNeuroscience	925	0.38
34	Molecular Systems Biology	822	0.34
35	Genome Medicine	676	0.28
36	Theoretical Biology and Medical Modeling	498	0.21
37	Comparative and Functional Genomics	466	0.19
38	Neuroinformatics	385	0.16
39	Cancer Informatics	355	0.15
40	Briefings in Functional Genomics & Proteomics	290	0.12
41	Evolutionary Bioinformatics	249	0.10
42	Algorithms for Molecular Biology	245	0.10
43	Journal of Biomedical Semantics	240	0.10
44	BioData Mining	149	0.06
45	EURASIP Journal on Bioinformatics and Systems Biology	140	0.06
46	Source Code for Biology and Medicine	131	0.05
Total		241,569	100.00

### Data preprocessing and keyphrase extraction

**Fig. 2:**
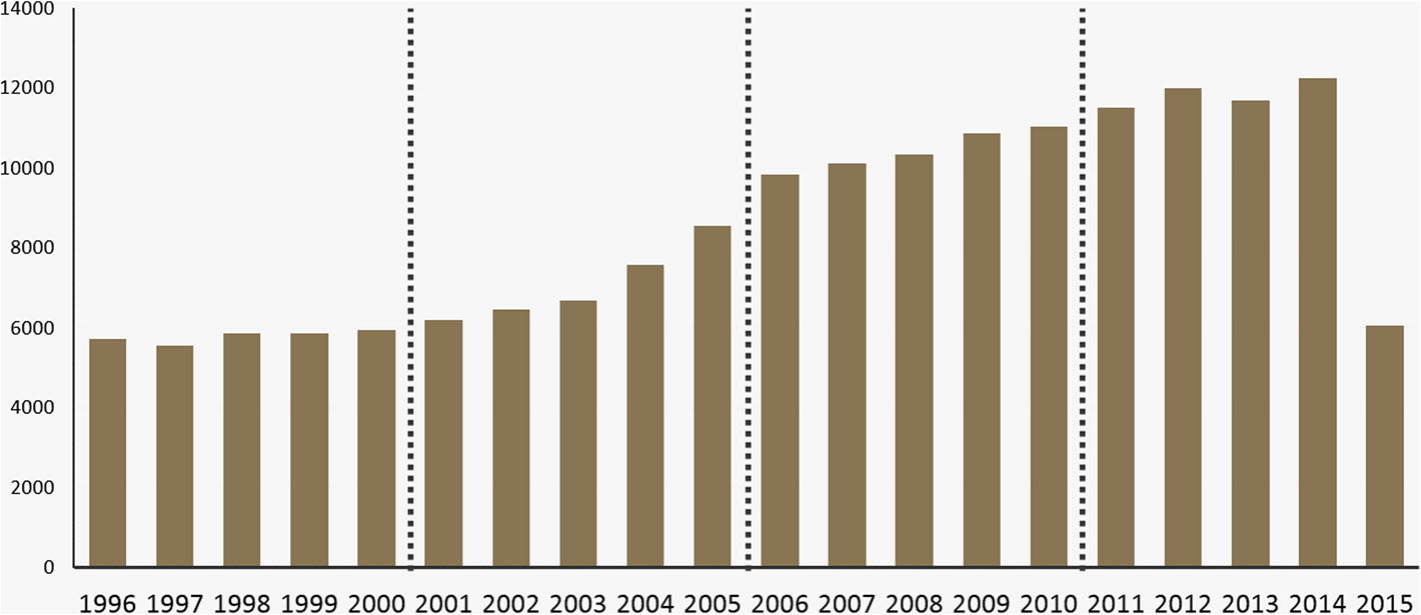
Data distribution. Publication year of our dataset is from 1996 to 2015. To identify topical trends of bioinformatics, we divided total 20 years into four time periods. X-axis is publication year and Y-axis is the number of papers

We limit the publication year back to 1996 and divide the dataset into the following four time periods to identify the trend of bioinformatics from the birth of the field to present: 1996–2000, 2001–2005, 2006–2010, and 2011–2015 (Fig. 2).

As shown in Fig. 2, there is a relatively consistent increase in the number of papers. There are fewer than half as many papers published in 2015 than in 2014 because we collect our dataset in June 2015. Nevertheless, we include the 2015 data to observe the latest publication trends. Table 2 presents the breakdown of our dataset by period. As in Fig. 2, the fourth period is the most productive, containing 53,520 papers, or 31.46% of the total dataset. The most productive year is 2014, which accounts for 7.20% with 12,251 papers. The total number of papers for all 20 years is 170,099. This number is different from Table 1 (241,569) as a result of preprocessing; we exclude papers that do not have an abstract.

**Table 2:** Time-based statistics for 20 years

Year	Number of Papers	Ratio (%)	Ranking
1996	5,713	3.36	19
1997	5,549	3.26	20
1998	5,853	3.44	18
1999	5,877	3.46	17
2000	5,947	3.50	16
Period 1	28,939	17.01	
2001	6,199	3.64	14
2002	6,456	3.80	13
2003	6,668	3.92	12
2004	7,564	4.45	11
2005	8,545	5.02	10
Period 2	35,432	20.83	
2006	9,845	5.79	9
2007	10,112	5.94	8
2008	10,352	6.09	7
2009	10,868	6.39	6
2010	11,031	6.49	5
Period 3	52,208	30.69	
2011	11,518	6.77	4
2012	11,986	7.05	2
2013	11,695	6.88	3
2014	12,251	7.20	1
2015	6,070	3.57	15
Period 4	53,520	31.46	
Total	170,099	100.00	

We extract various metadata, such as the PMID, author, publication year, journal title, title, and abstract, from XML formatted records. After XML processing, we combine the title with abstract and conduct keyphrase extraction. For keyphrase extraction, we use MAUI, which has the keyphrase model trained with MeSH terms [28]. In this dataset, there are 500 documents and several keys consisting of MeSH terms about each documents, which were manually assigned by the indexer. MAUI is a newer version of the keyphrase extraction algorithm KEA [29]. Keyphrase extraction enables researchers to select representative phrases to make topic detection more meaningful. Therefore, we use keyphrases extracted from the title or abstract as our input for the ACT model instead of individual words.

Table 3 shows the results of keyphrase extraction and other metadata such as the title and publication year from the PubMed record PMID 26030820.

**Table 3:** Example of results of keyphrase extraction and other metadata from PMID of 26030820

Information	Content
Title	encoding cell amplitude frequency modulation
Author	Micali Gabriele, Aquino Gerardo, Richards David M, Endres Robert G
Year	2015
Journal	PLOS computational biology
Keyphrases	Down-Regulation | Ion Channels | Ions | L Cells (Cell Line) | Ligands | Social Control, Formal | Social Control, Informal | Up-Regulation

### ACT Model Application

The ACT model, proposed by Tang et al. [17] as an extension of the LDA model [25], is a unified topic model for modeling various metadata simultaneously. This model starts with the assumption that the order of the topic created by the paper, author, and conference is same. It also estimates the statistical distribution associated with all topics for the purpose of discovering latent topic distribution related with paper, author, and conference. In this paper, two metadata types are changed. First, conference is replaced with journal. Also, a bag-of-keyphrases are used instead of a bag-of-words to represent documents in a more precise manner.

**Fig. 3:**
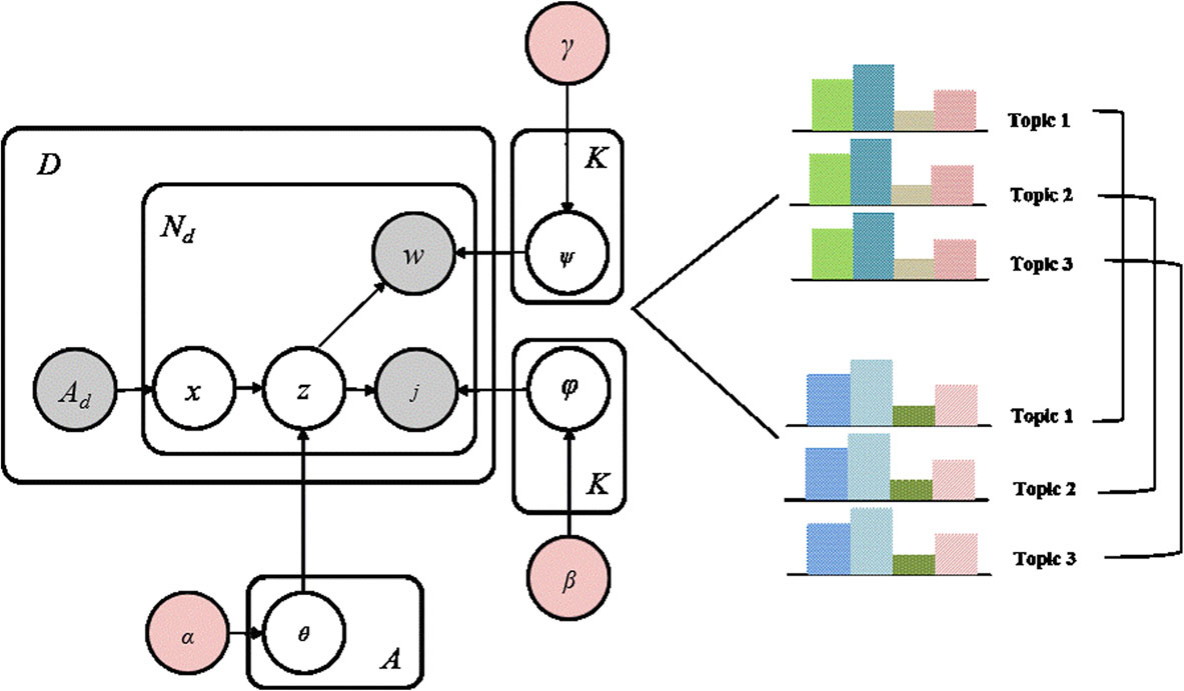
ACT Model. Author-Conference-Topic (ACT) Model is proposed by Tang et al. which is a probabilistic topic model to extract topics, authors, and conference simultaneously

Figure 3 illustrates the ACT model, and Table 4 provides a description of the parameters used. Model estimation is conducted by setting parameters, and for estimation of the model parameter, the Gibbs sampling method is employed. Gibbs sampling takes samples from a probability distribution by using Markov Chain Monte Carlo sampling method. Three parameters for estimating the model are as follows: 1) θ is the topic probability for a given author (author*topic matrix), 2) φ is the journal probability for a given topic (topic*journal matrix), 3) ψ is the word probability for a given topic (topic*word matrix). According to the independence assumption, joint distribution of topic, author, journal, and word stand on the basis A_d_, meaning the total number of authors in paper d. In our experiments, we set the hyper-parameters, α, β, γ, which are parameters of a prior with *α* = 50/T, *β* = 0.01, and γ = 0.01, respectively. In addition, we fix the number of topics K to 20, the number of top keyphrases to 30, the number of iterations to 1,000. With these settings, we selected 15 out of 20 topics for analysis.

**Table 4:** Notation and description of the ACT model

d	Paper	N_d_	Total number of words in paper d
x	Author	A_d_	Total number of authors in paper d
w	Word	z	Topic
j	Journal	θ	Author-topic distribution
D	Total number of papers	φ	Topi**c**-journal distribution
A	Total number of authors	ψ	Topic-word distribution
K	Selected number of topics	α,β,γ	Hyper-parameters of Dirichlet distribution

### Evaluation

To examine consistency of our results, we repeated each run 10 times with a topic number of 20. After that, we calculated the similarity between topics. For statistical analysis, we compute Pearson correlation coefficients between any two topics and average them out. Table 5 shows the average of correlation coefficients per execution. In all runs, Pearson correlation coefficients between topics were weakly, positively correlated. Also, the range of correlation was not wide (0.13 to 0.18). It implies that there was no difference in similarity between topics regardless of different runs. This result can verify consistency and reliability of our topic clusters.

**Table 5:** Average of Pearson correlation coefficients result

Number of Runs	Pearson correlation coefficients
1	0.155
2	0.140
3	0.152
4	0.177
5	0.180
6	0.146
7	0.136
8	0.160
9	0.158
10	0.178

**Fig. 4:**
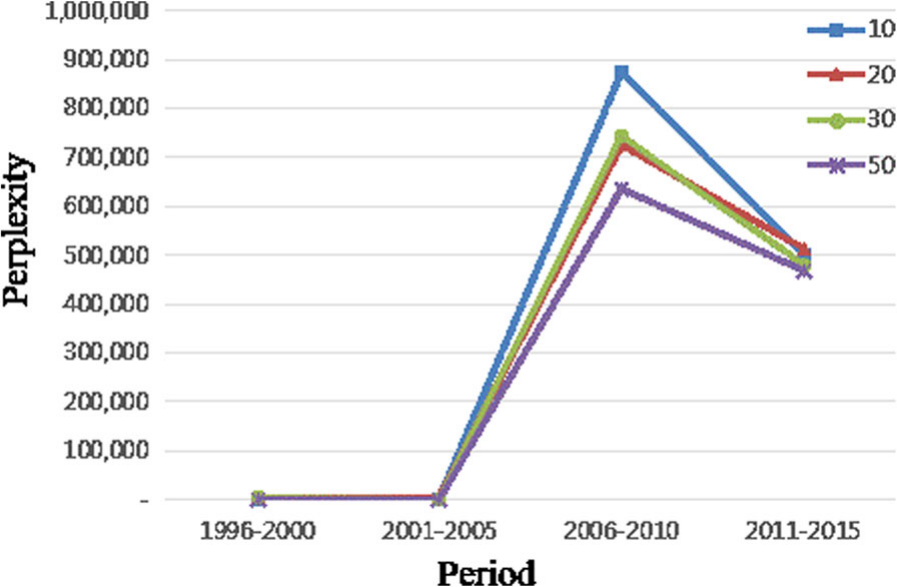
Perplexity result. For evaluation of topic modeling results, we used perplexity. We calculated perplexity per each period with the number of topics as 10, 20, 30, and 50. X-axis is period and Y-axis means a perplexity value

In addition, to evaluate the topic model results, we used perplexity which is a well-known measurement in information theory for testing goodness of a model. In our case, we make a test set by collecting bioinformatics journals published in 2016. The sample size is 1,000 papers. In the training set, we divided 20 years into 4 periods and calculated the perplexity by setting the number of topics as 10, 20, 30, and 50 respectively. The results are presented in Table 6 and Fig. 4. As shown in Table 6 and also confirmed in Fig. 4, there is not much difference in performance in regards to the number of topics by perplexity. However, there is a clear difference among periods by perplexity. In particular, the 3^rd^ period has the highest perplexity value, which implies that it is the most difficult period as to predicting the topic trend in 2016 in the bioinformatics field.

**Table 6:** Perplexity result of topic model

Number of Topics	1996–2000	2001–2005	2006–2010	2011–2015	Average
10	2,712	2,060	875,088	501,176	345,259
20	2,978	3,161	726,329	513,176	311,411
30	2,872	2,176	742,307	481,875	307,308
50	2,480	2,149	635,960	466,676	276,816
Average	2,760	2,387	744,921	490,726

Together with this result, we analyzed the results of the ACT model.

## Results

We analyze leading authors and journals in relation to topics over time. In the following section, we provide the detail explanations of the trend per period.

### Topic analysis per period

The results of our time series topic analysis show that topics seem to be more distinct and subdivided closer to present. In addition, new topics have emerged in recent years, and they do not make a new cluster, which means the exclusive topics become apparent. The results also show that research fields such as molecular biology, genomics, genetics, and proteomics play a supplementary role in biology, but also become diversified into a unique field.

#### First period analysis

In the first period (1996–2000), five dominant topic clusters are identified (Additional file 1: Appendix 1). Those five topics are mainly associated with proteins and peptides. Phrases such as molecular biology and chemical compound are widespread, and thermodynamics- and kinematics-related topics appear. These topics are composed of jargon in their specific fields. The mathematical biology field is shown by topical phrases such as database, cluster analysis, model, theoretical, and software.

Topics 0, 2, and 3 are about molecular biology, which are derived from biochemistry and composed of hydrogen bonding–related chemical compounds such as enzymes or lipids. Topics 4, 5, 6, and 7 are related to proteins, peptides, and protein structure. Topics 9 and 14 include words such as ‘probability’ and ‘statistics’, which are related to mathematical biology. Topics 13, 17, 18, and 19 cover mutagenesis, disease, and syndromes. These are all related with genetic diseases. Mutagenesis consists of gene mutation, and syndromes are caused by genetic disorder. Topic 19 includes the word ‘genetic’ that is a parent category of previously mentioned words. Topics 15 and 16 consist of kinetics.

Protein-related topics are dominant, and authors involved in peptide and protein structure are prevalent in the first period. Authors who are in topic 5, such as Fersht A.R., Thornton J. M., Dobson C. M., Serrano L. and Karplus M., have a high probabilistic distribution value, which means they are leading researchers in this area. Their research interest is mainly in protein structure, and they have publications in the Journal of Molecular Biology. This journal appears in almost all of the topics related to protein and deals with structure and function of macromolecules, complexes, and protein folding.

#### Second period analysis

There are four topic clusters and one exclusive topic in the second period (Additional file 1: Appendix 2). In the second period (2001–2005), studies about genetics and genomics are actively conducted, and protein-related topics are diversified into subfields such as proteomics. In addition, mathematical biology and computational biology–related topics are maintained in this period.

Topics 1, 2, 5, 7, and 11 include DNA mechanism, molecular structure, genetics, genomics, and diseases caused by DNA or genome such as Down syndrome, DNA transposable elements, and ribonucleases. Topics 0, 3, 14, and 16 are mainly about proteomics, specifically focusing on protein structure. Topics 12, 18, and 19 contain biotechnology, molecular modeling, and structure. Topics 8 and 9 focus on mathematical biology and computational biology. Topic 4 exclusively contains enzymology-related phrases such as enzyme activators and oxygen. Enzymology-related topics are less common compared with the first period.

The second period mainly focuses on gene-related topics. Topic 5 has the highest probabilistic distribution among top-ranked authors such as Petsko Gregory A., Aravind L., Koonin Eugene V., Gerstein Mark., and Hurst Laurence D. They are interested in genomics and biomedical engineering. Those authors publish papers in Genome Biology. Genome Biology covers subject matters related to genomics and post-genomics. Similar to the first period, protein-related research is a major topic in the second period. Top-ranked authors in this topic include Aebersold Ruedi, Roepstorff Peter, Righetti Pier Giorgio, Sanchez Jean-Charles, and Jungblut Peter R. These authors are pioneers of proteomics. Their papers are published in the Journal of Molecular Biology and Proteomics.

#### Third period analysis

In the third period (2006–2010), the topics are divided into three clusters: genomics, proteomics, and other (Additional file 1: Appendix 3). Different from the first two periods, four exclusive topics exist and seem to be distinct from topics in the other three periods. For instance, studies about genomics or proteomics are more diversified than in the earlier periods. Exclusive topics that are not included in two large fields emerge, indicating that bioinformatics research is conducted in various fields related to bioinformatics.

Topics 3, 7, 10, 11, 13, and 16 consist of proteomics, protein evolution, and protein structure. Proteomics-related topics are subdivided. The representative journals in the area are Proteomics, the Journal of Proteome Research, and the Journal of Proteomics. Topics 5, 6, 12, 14, and 19 are gene-related topics such as gene expression, gene transcription, and genomics. Gene-related studies become prevalent in the second period. The distinct topics that appear in the third period are topics 0, 15, 17, and 18. Topic 0 is about molecular biology, especially focusing on hydrogen bonding. In the first and second periods, topic 15 includes various topics related to theoretical biology. Topic 17 is related to hepatitis, the infection in liver cells and tissues. Different from previous periods, in the third period topics are associated with specific diseases. Topic 18 includes peptide-associated phrases, and, unlike prior periods, concrete themes like specific chemical compounds and protein appear.

Overall, protein-related topics are most common in the third period. The third period also has more sub-divided and distinct topics than previous periods do. In this period, general topics such as proteomics appear, as do specific topics such as protein evolution, protein analytics, and protein ubiquitin. Among these areas, the topic with the highest distribution is analytics about protein, and it is sub-categorized in proteomics. Top-ranked authors in this period include Mann Matthias, Aebersold Ruedi, Smith Richard D., Heck Albert J. R., and Thongboonkerd Visith. They are experts in protein analytics, and commonly use mass spectrometry for their analyses. They actively publish in the Journal of Proteome Research and Proteomics. These two journals are top-rated journal in protein-related topics. The Journal of Proteome Research is computer technology–oriented and focused on protein-analysis research. The journal with the highest probabilistic distribution in all topic areas is the EMBO Journal. This journal is focused on molecular biology and also covers proteomics.

#### Fourth period analysis

The fourth period (2011–2015) shows three major topic clusters and two exclusive topics (Additional file 1: Appendix 4). Similar to the third period, the topics related with genomics and proteomics are further divided into subfields and represent concrete topical characteristics. Compared with the third period’s results, theoretical biology–related topics form one cluster. The compositions of the cluster are one big topic (systems biology) and four sub-divided topics.

Topics 1 and 16 are theoretical biology–related, and topics 6 and 10 are about systems biology. They can be clustered as a broader category of system biology. The representative journals in this cluster are PLOS Computational Biology, Journal of Theoretical Biology, and Journal of Computational Neuroscience, which are focused on systems biology. Topics 0, 11, 12, 18, and 19 are about genetics and genomics. Topics 4, 9, 13, and 17 represent proteomics. Exclusive topics are topics 8 and 15, each of which is related to molecular biology and cell biology. Topic 8 includes phrases like hydrogen bonding, and GTP-binding proteins, and topic 15 contains phrases like enteroendocrine cells and COS cells. The top journals in these areas are biochemistry, journal of molecular biology, and journal of molecular modeling.

In the fourth period, the major topics are systems biology, genomics, and proteomics. Topics that are not in the main stream of bioinformatics are found in this period, and topics about theoretical biology and systems biology become a distinct cluster. This means that these areas are growing in the bioinformatics area. The representative researchers in this area are Nowak Martin A., Iwasa Yoh, Steel Mike, Dieckmann Ulf, and Paninski Liam. They are mostly involved in mathematics and theoretical biology. The journal which has the highest probabilistic distribution is the Journal of Theoretical Biology. This journal is focused on research that combines biology and topics such as statistical analysis, mathematical definition, comparative research, experiment, and computer simulation. The second raked the Journal of Bioinformatics, which mainly accepts research about genome bioinformatics and computational biology.

## Discussion

In this section, we analyzed the results from three different perspectives: topical keyphrase, journal, and author. In addition, to further identify which researchers and journals focus on which topic over time, the results of the ACT model (top-ranked keyphrases, authors, and journals) are examined in an integrated perspective.

### Time series analysis

One interesting observation is that keyphrases related with genes or genetic processes such as ‘gene expression’, ‘down-regulation’, and ‘up-regulation’ were not ranked high in the first period. However, they emerged as top keyphrases in later periods. In particular, ‘proteome’, ‘reproducibility of results’, ‘proteomics’, and ‘genotype’ did not appear in the first period but emerged gradually after that. In addition, the term ‘algorithms’ appeared most frequently in the fourth period. In author perspective, across the four periods, the number of unique authors was 1,396. Top ranked author Robinson Richard appeared in five topics. Seven authors, including Gross Liza, appeared four times, 45 authors appeared three times, 137 authors appeared twice, and the remaining 1,184 authors were shown only in one topic. There was no author who appears in all four periods. Thirty-nine authors appeared across three periods, 125 authors appeared in two periods, and 1,210 authors appeared only in a period. In journal centered view, only 21 out of 46 journals appeared in the first period. In the second and third period, 34 and 46 journals were presented respectively. Forty-five journals appeared in the last period; one the journal ‘Briefings in Functional Genomics & Proteomics’ was not shown in the last period.

These results imply that the bioinformatics field is diversified and new topical disciplines are recently emerged. For instance, proteomics-related topics start to appear in the second period, become segmented into detail research fields and later evolved in the third and fourth periods. In addition, while conceptual biology–related topics exist in the first period, they become clearly progressed in the fourth period. Conversely, the topics about kinetics appear in the first period, but then fade out.

### Integrated view of graph pattern analysis

**Fig. 5:**
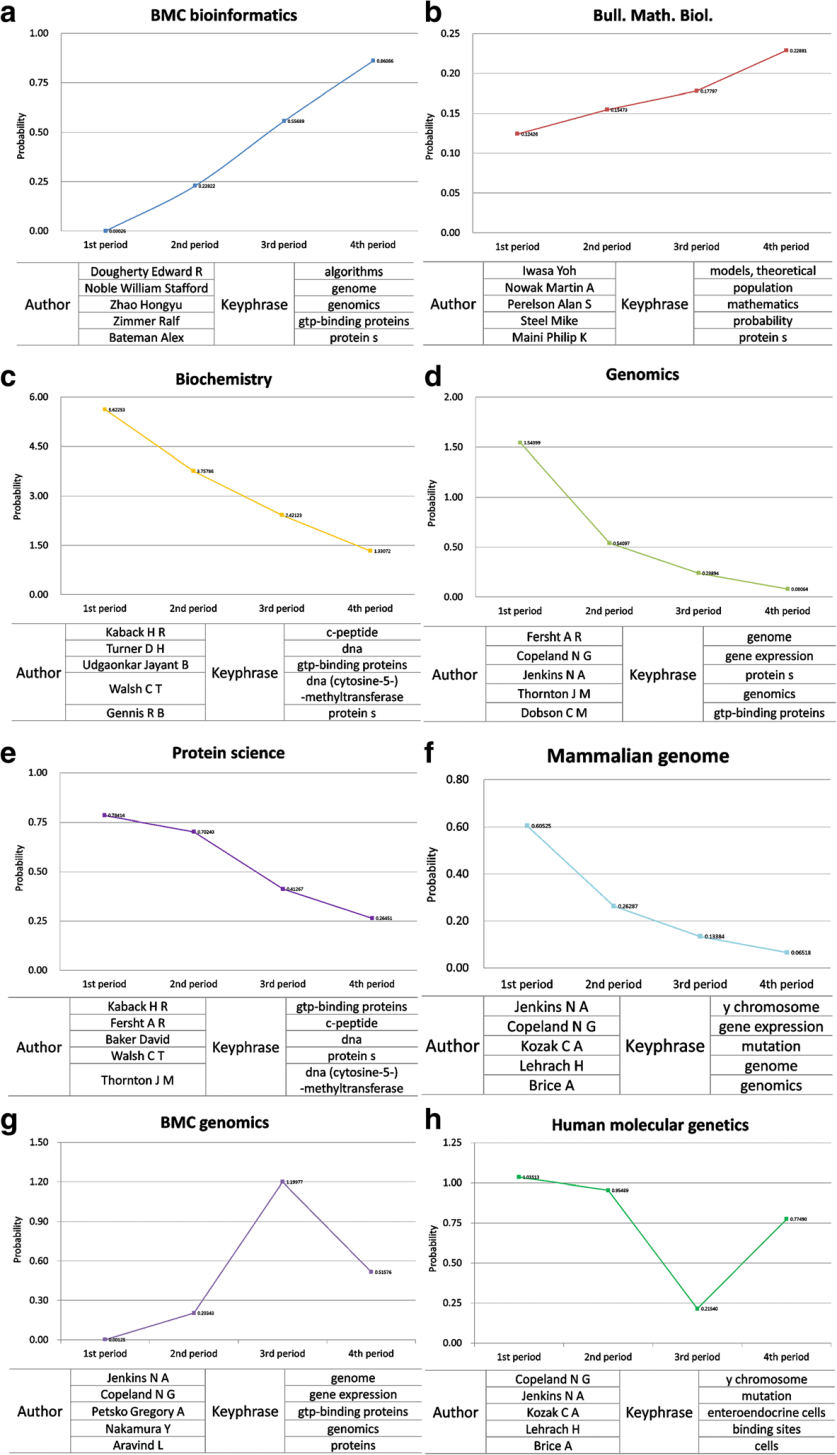
Journal focused topic distribution with related authors and keyphrases. For integrated pattern analysis, we examined eight representative journals with top authors and keyphrases. Patterns were classified as four outstanding ones such as rising (**a**-**b**), falling (**c**-**f**), convex ﻿(**g**) and concave (**h**)

For further integrated analysis, we examined top journals with their topical probability in all 4 periods. We also checked the authors and topical keyphrases which were topically matched with the journals. We identified that there were four different patterns in journal’s topical distribution such as rising, falling, concave, and convex pattern. In Fig. 5, we only presented graphs which drastically changed in terms of the probability value of topics between periods. Additionally in each graph, we presented top 5 ranked authors and keyphrases which have a high probability value across 4 periods.

We explained four outstanding cases in each patterns and made a list of journals which are showed in all four periods (Table 7). First for the rising pattern, the journal ‘BMC Bioinformatics’ had 0.86060 gaps between maximum and minimum probability. It was the highest gap by probability in all rising patterns. The average impact factor of this journal provided by journal citation report (JCR) was 3.0806 in 2015. In this context, BMC Bioinformatics could be regarded as the promising journal in the bioinformatics field. The journal has grown steadily through 20 years. The authors belonging to the similar topical scope of BMC Bioinformatics were presented in graph (a). The top ranked authors shared common characteristics. The authors were majored in computer science or statistics and then applied the technique to biomedical or biology area later on. Their common research interests were bioinformatics or biostatistics. As shown in graph (a), the topical keyphrases related with the journal were not focused only on biology research fields. The word ‘algorithms’ represents the informatics field, ‘genome’, ‘genomics’, ‘gtp-binding proteins’ means genomics related fields and the word ‘protein s’ represents protein or gene related scopes. The scope of the journal is in computational and statistical methods for the modeling and analysis of all kinds of biological data, as well as other areas of computational biology. The result indicated that the publication trend of BMC Bioinformatics changed to genetics or genomics converged with informatics. In case of the falling pattern of journal ‘Biochemistry’, as shown in graph (c), the journal had 4.29181 gap between the maximum and minimum value. The average impact factor of this journal in 4 periods was 3.75322. The journal had somewhat a high probability value, but the impact factor in each periods decreases gradually (e.g., 1^st^ period: 4.4785 to 4^th^ period: 3.1768). This decreasing pattern implied that in bioinformatics field, the journal dealt mainly with biochemistry, biophysical chemistry, and molecular biology, but it was not converged with informatics. That means the biochemistry field had not much interaction with informatics fields. The top ranked authors shown in graph (c) were celebrated scholars in biochemistry field. Commonly, they all researched only the biochemistry field and we could not find that they had the connection with the informatics area. At the same context, top ranked keyphrases of the journal were mostly related to biochemistry research fields. The informatics related keyphrase did not appear in the top word list.

**Table 7:** The list of journals showed in all periods

Journal Name	Sum of Probability	Average of Probability
Biochemistry	13.13233	3.28308
Bioinformatics (Oxford, England)	2.49214	0.62304
BMC Bioinformatics	1.64624	0.41156
BMC Genomics	1.92022	0.48005
Bulletin of mathematical biology	0.68576	0.17144
Genome biology	1.28690	0.32173
Genomics	2.40453	0.60113
Human molecular genetics	2.98031	0.74508
Journal of biotechnology	1.32392	0.33098
Journal of computational neuroscience	0.42877	0.10719
Journal of computer-aided molecular design	0.60322	0.15081
Journal of molecular biology	5.82534	1.45633
Journal of theoretical biology	3.00820	0.75205
Mammalian genome	1.06714	0.26678
Physiological genomics	0.61599	0.15400
Protein science	2.16372	0.54093
Statistical methods in medical research	0.25062	0.06266
The EMBO journal	3.35207	0.83802
Trends in biochemical sciences	0.50571	0.12643
Trends in biotechnology	0.50871	0.12718
Trends in genetics	0.73352	0.18338

Not only the rising and falling patterns, but the convex and concave patterns of journals exist. In graph (g), BMC Genomics was the journal showing the convex pattern. BMC Genomics dealt with genome-scale analysis, functional genomics, and proteomics. The average impact factor from 2000 to 2015 was 4.0464. The topical probability of the journal rose from 1^st^ period (0.00125) to 3^rd^ period (1.19977), but since then the probability dropped to 0.51576. The gap between maximum and minimum value was 1.19852. The journal changed the status to open access in 2000, and maybe it caused the slight rise from 1^st^ period to 2^nd^ period. For the drastic increase from 2^nd^ period to 3^rd^ period, the number of total citations increased approximately 2 times according to the JCR. The increasing and decreasing number in total citations may cause the wave in graph (g). The top 5 ranked authors’ research interests had various characteristics. Jenkins N.A and Copeland N.G was a couple. Jenkins N.A was interested in molecular & cellular biology, while the husband Copeland N.G was in the biochemistry field. They collaborated a lot and both were celebrated researchers in the field. The other researchers had also different research areas; treatment or preventive therapy (Petsko Gregory A), genomic medicine (Nakamura Y), and genome analysis (Aravind L). Through all 4 periods, the journal mainly published articles which dealt with the genomic related keyphrases. The authors and keyphrases located in this journal were not quite related to the informatics fields. Last, the concave pattern was shown in graph (h), and Human molecular genetics was the representative journal. The journal had steadily decreasing impact factor flow in 20 years (e.g., 1^st^ period: 9.05475 to 4^th^ period: 6.8766). The gap between maximum and minimum probability was 0.81973. In graph (h), the drastic fallen point exists. Top ranked authors related with the journal focused on genetics. Copeland N.G and Jenkins N.A appeared again. Rest of the authors did not overlap in research areas. The journal was interested in broad genetics related topical keyphrases but not related with informatics fields.

Through the pattern analysis integrated with journal, author, and keyphrase, we identified that the bioinformatics field was a converging area, and certain journals clearly showed rising and falling patterns. Different from period analysis, the integrated view of analysis showed journals’ topical trends over time along with top journals and researchers.

## Conclusion

Bioinformatics mainly tackles biological problems at the molecular level using applied mathematics, information science, statistics, computer science, chemistry, and biochemistry. This characteristic of bioinformatics has driven the field to become interdisciplinary, combining approaches from various fields to make use of a large amount of data.

In this study, we investigate the bioinformatics field using the ACT model to conduct comprehensive topic analyses of keyphrases, authors, and journals. To this end, we collect 46 journals belonging to the bioinformatics field by searching journal name in PubMed, yielding 170,099 papers. To analyze topic evolution over time, we divide the collected datasets into four, five-year periods: 1996–2000, 2001–2005, 2006–2010, and 2011–2015. In a time series topic analysis, we examine topic clusters within period. In more recent periods, distinct characteristics of the field emerge and more new topics are presented independently. In addition, we analyze trends in keyphrases, authors, and journals. Our keyphrase analysis similarly indicates the emergence of greater interdisciplinary research over time. In our author analysis, we observe the pattern of authors who appear in top rank. In our journal analysis, we analyze the common topic area of top journals and identify major focus areas of those top journals, including computational biology, theoretical biology, and mathematical biology. Also, we examine topic distribution over journal with top ranked authors and keyphrases. In this analysis, journals are identified as four patterns over time such as rising, falling, convex, and concave patterns.

The results of these analyses imply that the bioinformatics field is highly interdisciplinary, consisting of active convergence studies. In addition, we observed that the characteristics of the bioinformatics field become more distinct and more specific, and the supporting role of peripheral fields of bioinformatics, such as conceptual, mathematical, and systems biology, gradually increases over time, though the core fields of proteomics, genomics, and genetics are still the major topics. This is consistently confirmed by analysis of topic distribution of journals over time as well as integrated analysis of top ranked keyphrases, authors, and journals.

In the future, we plan to apply the same approach to other domains, such as information science. We also plan to use other metadata such as MeSH terms and bio-entities to compare with keyphrases. In addition, we plan to explore how to infer authors’ interests by time series analysis and identify representative authors and the journals that are the best suited for a paper on a particular subject.

## Additional file

Additional file 1: Appendix 1. ACT model results in first period. Appendix 2. ACT model results in second period. Appendix 3. ACT model results in third period. Appendix 4. ACT model results in fourth period. (DOCX 48 kb)
