# Supplementary material for: Analyzing the field of bioinformatics with the multi-faceted topic modeling technique
Source: BMC Bioinformatics. 2017 May 31;18(Suppl 7):251. doi: 10.1186/s12859-017-1640-x (PMC5471940; doi:10.1186/s12859-017-1640-x)
Supplement: Additional file 1: — Appendix 1. ACT model results in first period. Appendix 2. ACT model results in second period. Appendix 3. ACT model results in third period. Appendix 4. ACT model results in fourth period. (DOCX 48 kb) [file 12859_2017_1640_MOESM1_ESM.docx]

**Additional file 1**

**Appendix 1. ACT Model results in first period**

| Topic 0 | | Topic 2 | | Topic 3 | | Topic 4 | |
| --- | --- | --- | --- | --- | --- | --- | --- |
| Enzyme,  Molecular Biology | | Lipid,  Molecular Biology | | Hydrogen Bonding,  Molecular Biology | | Protein Binding,  Protein structure | |
| escherichia coli | 0.09041 | Lipids | 0.02436 | Oxidants | 0.05985 | binding sites | 0.08011 |
| Metals | 0.02777 | lipid a | 0.02313 | oxidoreductases | 0.05258 | apolipoproteins a | 0.04669 |
| enzyme activators | 0.02173 | membranes | 0.02313 | Oxides | 0.05068 | apolipoproteins e | 0.04627 |
| aspartic acid | 0.02147 | phospholipids | 0.01565 | hydrogen bonding | 0.03183 | apolipoproteins c | 0.04595 |
| enzyme activation | 0.01954 | phospholipases a | 0.01073 | Oxygenators | 0.02188 | apolipoproteins b | 0.04319 |
| Kaback H R | 0.00090 | Jain M K | 0.00030 | Gennis R B | 0.00041 | Moras D | 0.00012 |
| Raushel F M | 0.00033 | Taylor S S | 0.00029 | Klinman J P | 0.00032 | Proudfoot N J | 0.00011 |
| Walsh C T | 0.00027 | Seefeldt L C | 0.00028 | Ballou D P | 0.00031 | Bass B L | 0.00011 |
| Rezaie A R | 0.00020 | Roberts M F | 0.00022 | Rutherford A W | 0.00031 | Gautel M | 0.00010 |
| Benkovic S J | 0.00018 | Wittinghofer A | 0.00018 | Marletta M A | 0.00026 | Neuberger M S | 0.00010 |
| Biochemistry | 0.85912 | Biochemistry | 0.97460 | Biochemistry | 0.83154 | The EMBO journal | 0.51000 |
| Protein science : a publication of the Protein Society | 0.13401 | Protein science : a publication of the Protein Society | 0.01337 | Journal of molecular biology | 0.06753 | Journal of molecular biology | 0.32921 |
| Journal of biotechnology | 0.00433 | Trends in biochemical sciences | 0.01134 | Protein science : a publication of the Protein Society | 0.04569 | Protein science : a publication of the Protein Society | 0.03604 |
| Journal of computer-aided molecular design | 0.00115 | Genomics | 0.00045 | The EMBO journal | 0.01967 | Genomics | 0.03219 |
| Journal of molecular biology | 0.00063 | Genome biology | 0.00010 | Journal of biotechnology | 0.01402 | Human molecular genetics | 0.02204 |
| Topic 5 | | Topic 6 | | Topic 7 | | Topic 9 | |
| Computational biology, Protein | | Peptides,  Protein structure | | Peptides,  Protein structure | | Cell,  Mathematical biology | |
| gtp-binding proteins | 0.10356 | dna | 0.10757 | c-peptide | 0.09222 | enteroendocrine cells | 0.07828 |
| protein s | 0.07393 | dna (cytosine-5-)-methyltransferase | 0.09950 | peptide t | 0.05378 | cells | 0.05449 |
| Proteins | 0.06786 | dna-(apurinic or apyrimidinic site) lyase | 0.06051 | peptides | 0.05321 | cos cells | 0.02895 |
| viral fusion proteins | 0.05970 | dna-directed rna polymerases | 0.02126 | peptide phi | 0.03950 | l cells (cell line) | 0.02145 |
| protein c | 0.05362 | dna polymerase i | 0.01328 | urea | 0.01352 | models, theoretical | 0.00856 |
| Fersht A R | 0.00085 | Turner D H | 0.00040 | Craik D J | 0.00030 | Sherratt J A | 0.00017 |
| Thornton J M | 0.00069 | Patel D J | 0.00032 | Walsh C T | 0.00025 | Mochizuki A | 0.00015 |
| Dobson C M | 0.00065 | Lilley D M | 0.00026 | Shai Y | 0.00017 | Kaneko K | 0.00012 |
| Serrano L | 0.00062 | Lippard S J | 0.00026 | Baldwin R L | 0.00017 | van Ooyen A | 0.00011 |
| Karplus M | 0.00051 | Herschlag D | 0.00019 | Cafiso D S | 0.00017 | Maini P K | 0.00010 |
| Journal of molecular biology | 0.64594 | Biochemistry | 0.51252 | Biochemistry | 0.87886 | The EMBO journal | 0.25633 |
| Protein science : a publication of the Protein Society | 0.35348 | Journal of molecular biology | 0.34588 | Protein science : a publication of the Protein Society | 0.05831 | Journal of theoretical biology | 0.16309 |
| Trends in biochemical sciences | 0.00030 | The EMBO journal | 0.10983 | Journal of molecular biology | 0.03032 | Biochemistry | 0.11381 |
| Genomics | 0.00008 | Trends in biochemical sciences | 0.01463 | Journal of computer-aided molecular design | 0.02906 | Journal of biotechnology | 0.07245 |
| Journal of theoretical biology | 0.00008 | Protein science : a publication of the Protein Society | 0.01184 | Genomics | 0.00117 | Journal of computational neuroscience | 0.06773 |
| Topic 13 | | Topic 14 | | Topic 15 | | Topic 16 | |
| Mutagenesis,  Chromosome | | Mathematical biology | | Kinase | | Kinetics | |
| y chromosome | 0.05293 | models, theoretical | 0.06474 | signal transduction | 0.03888 | kinetics | 0.11937 |
| Exons | 0.03035 | population | 0.01891 | proto-oncogene proteins c-akt | 0.03348 | mutation | 0.07270 |
| chromosome mapping | 0.02731 | mathematics | 0.01025 | phosphorylation | 0.02499 | dimerization | 0.03195 |
| Introns | 0.02035 | probability | 0.00919 | protein kinase c | 0.02489 | tryptophan | 0.02973 |
| physical chromosome mapping | 0.01598 | statistics | 0.00887 | protein-tyrosine kinase | 0.01949 | energy transfer | 0.01593 |
| Jenkins N A | 0.00061 | Nowak M A | 0.00041 | Schlessinger J | 0.00019 | Turner D H | 0.00017 |
| Copeland N G | 0.00060 | Perelson A S | 0.00033 | Hunter T | 0.00015 | Squier T C | 0.00017 |
| Nakamura Y | 0.00033 | Frank S A | 0.00025 | Davis R J | 0.00014 | Davidson V L | 0.00016 |
| Kozak C A | 0.00026 | Iwasa Y | 0.00022 | Hemmings B A | 0.00014 | Thomas D D | 0.00012 |
| Scherer S W | 0.00025 | Maini P K | 0.00014 | Jenkins N A | 0.00011 | Kaback H R | 0.00012 |
| Genomics | 0.83768 | Journal of theoretical biology | 0.63340 | The EMBO journal | 0.61168 | Biochemistry | 0.96262 |
| Mammalian genome : official journal of the International Mammalian Genome Society | 0.13170 | Journal of computer-aided molecular design | 0.12867 | Genomics | 0.10066 | Protein science : a publication of the Protein Society | 0.02313 |
| Human molecular genetics | 0.02175 | Bulletin of mathematical biology | 0.08536 | Biochemistry | 0.08476 | Journal of biotechnology | 0.00579 |
| Physiological genomics | 0.00738 | Statistical methods in medical research | 0.07227 | Journal of molecular biology | 0.05628 | Journal of computer-aided molecular design | 0.00364 |
| BMC genomics | 0.00073 | Journal of computational neuroscience | 0.03343 | Trends in biochemical sciences | 0.03997 | Trends in biochemical sciences | 0.00262 |
| Topic 17: | | Topic 18: | | Topic 19 | |  |  |
| Virus ,  Genetics | | Gene related Disease, Genetics | | Genetics | |  |  |
| recombination, genetic | 0.02550 | syndrome | 0.02348 | mutation | 0.02448 |  |  |
| escherichia coli | 0.02315 | down syndrome | 0.02264 | genome | 0.01926 |  |  |
| recombinant proteins | 0.01056 | y chromosome | 0.01972 | genomics | 0.01148 |  |  |
| Yeasts | 0.00928 | family | 0.01700 | genetic markers | 0.01091 |  |  |
| Bacteria | 0.00875 | mutation, missense | 0.01456 | polymorphism, genetic | 0.01053 |  |  |
| Lubitz W | 0.00022 | Brice A | 0.00038 | Lehrach H | 0.00047 |  |  |
| Bjorkman P J | 0.00017 | Davies K E | 0.00029 | Kozak C A | 0.00032 |  |  |
| Withers S G | 0.00011 | Ballabio A | 0.00028 | Copeland N G | 0.00025 |  |  |
| Dijkstra B W | 0.00011 | Jeffreys A J | 0.00025 | Davisson M T | 0.00020 |  |  |
| De Francesco R | 0.00011 | Peltonen L | 0.00025 | Barendse W | 0.00020 |  |  |
| Biochemistry | 0.38659 | Human molecular genetics | 0.81524 | Genomics | 0.42037 |  |  |
| Journal of biotechnology | 0.28780 | Genomics | 0.11735 | Mammalian genome : official journal of the International Mammalian Genome Society | 0.40967 |  |  |
| Journal of molecular biology | 0.15256 | Mammalian genome : official journal of the International Mammalian Genome Society | 0.02374 | Human molecular genetics | 0.09421 |  |  |
| Protein science : a publication of the Protein Society | 0.06838 | Trends in genetics : TIG | 0.01911 | Trends in genetics : TIG | 0.03373 |  |  |
| Trends in biotechnology | 0.04097 | The EMBO journal | 0.00956 | Physiological genomics | 0.01472 |  |  |

**Appendix 2. ACT Model results in second period**

| Topic 0 | | Topic 1 | | Topic 2 | | Topic 3 | |
| --- | --- | --- | --- | --- | --- | --- | --- |
| Protein structure,  Proteomics | | Gene related Disease, Genetics | | DNA mechanism,  Genetics | | Proteomics | |
| c-peptide | 0.10855 | mutation | 0.04925 | Dna | 0.08385 | proteome | 0.09386 |
| peptide t | 0.07003 | syndrome | 0.02011 | dna (cytosine-5-)-methyltransferase | 0.07565 | proteomics | 0.09121 |
| Peptides | 0.06973 | down syndrome | 0.01675 | dna-(apurinic or apyrimidinic site) lyase | 0.04435 | gtp-binding proteins | 0.07251 |
| peptide phi | 0.05397 | genotype | 0.01384 | dna transposable elements | 0.02273 | proteins | 0.05459 |
| hemoglobin m | 0.00769 | polymorphism, genetic | 0.01155 | dna-directed dna polymerase | 0.01662 | protein s | 0.04548 |
| Shai Yechiel | 0.00022 | Peltonen Leena | 0.00017 | Stivers James T | 0.00011 | Aebersold Ruedi | 0.00041 |
| Ho Chien | 0.00013 | Olson James M | 0.00010 | Geacintov Nicholas E | 0.00010 | Roepstorff Peter | 0.00027 |
| de Kruijff Ben | 0.00009 | Rubinsztein David C | 0.00010 | Berdis Anthony J | 0.00010 | Righetti Pier Giorgio | 0.00026 |
| Walsh Christopher T | 0.00009 | Eng Charis | 0.00009 | Halford Stephen E | 0.00010 | Sanchez Jean-Charles | 0.00025 |
| Segrest Jere P | 0.00009 | Zoghbi Huda Y | 0.00009 | Osheroff Neil | 0.00010 | Jungblut Peter R | 0.00023 |
| Biochemistry | 0.61368 | Human molecular genetics | 0.76183 | Biochemistry | 0.39844 | Proteomics | 0.60016 |
| Journal of molecular biology | 0.18197 | Genomics | 0.08126 | Journal of molecular biology | 0.37999 | Journal of proteome research | 0.20998 |
| Protein science : a publication of the Protein Society | 0.11657 | Pharmacogenetics and genomics | 0.04033 | The EMBO journal | 0.12732 | Molecular & cellular proteomics : MCP | 0.17391 |
| The EMBO journal | 0.02834 | Mammalian genome : official journal of the International Mammalian Genome Society | 0.02863 | Protein science : a publication of the Protein Society | 0.02130 | Briefings in functional genomics & proteomics | 0.01128 |
| Statistical methods in medical research | 0.00744 | Physiological genomics | 0.02355 | Trends in biochemical sciences | 0.01665 | Cancer informatics | 0.00273 |
| Topic 4 | | Topic 5 | | Topic 7 | | Topic 8 | |
| Enzymology,  Molecular Biology | | Gene expression,  Genetics | | Molecular structure,  Genetics | | Mathematical biology | |
| Oxidants | 0.04419 | genome | 0.04670 | ribonuclease, pancreatic | 0.01820 | models, theoretical | 0.05783 |
| Oxides | 0.04181 | genomics | 0.04087 | ribonuclease h, calf thymus | 0.01636 | population | 0.01905 |
| oxidoreductases | 0.03615 | gene expression | 0.02533 | protein kinases | 0.01336 | evolution | 0.01485 |
| enzyme activators | 0.01971 | down-regulation | 0.01000 | ribonuclease p | 0.01336 | mathematics | 0.01011 |
| Oxygen | 0.01466 | up-regulation | 0.00957 | ribonucleases | 0.01321 | probability | 0.00832 |
| Ballou David P | 0.00019 | Petsko Gregory A | 0.00041 | Taylor Susan S | 0.00019 | Iwasa Yoh | 0.00045 |
| Klinman Judith P | 0.00014 | Aravind L | 0.00027 | Huber Robert | 0.00014 | Nowak Martin A | 0.00024 |
| Fitzpatrick Paul F | 0.00010 | Koonin Eugene V | 0.00027 | Smith David L | 0.00012 | Perelson Alan S | 0.00014 |
| Scrutton Nigel S | 0.00010 | Gerstein Mark | 0.00024 | Raines Ronald T | 0.00011 | Kaneko Kunihiko | 0.00013 |
| Tu Shiao-Chun | 0.00010 | Hurst Laurence D | 0.00020 | Acharya K Ravi | 0.00009 | Maini P K | 0.00012 |
| Biochemistry | 0.79524 | Genome biology | 0.42328 | Biochemistry | 0.47160 | Journal of theoretical biology | 0.66880 |
| Journal of molecular biology | 0.06953 | Trends in genetics : TIG | 0.27688 | Journal of molecular biology | 0.24608 | Bulletin of mathematical biology | 0.14119 |
| Protein science : a publication of the Protein Society | 0.03316 | PLoS biology | 0.10465 | Protein science : a publication of the Protein Society | 0.11883 | Journal of computational neuroscience | 0.09321 |
| The EMBO journal | 0.02762 | BMC genomics | 0.09314 | The EMBO journal | 0.04938 | PLoS biology | 0.04571 |
| Trends in biochemical sciences | 0.01370 | Briefings in functional genomics & proteomics | 0.04053 | Journal of biotechnology | 0.01901 | Theoretical biology & medical modelling | 0.02186 |
| Topic 9 | | Topic 11 | | Topic 12 | | Topic 14 | |
| Computational biology | | Genetics | | Molecular modeling,  Molecular Biology | | Protein structure,  Proteomics | |
| Algorithms | 0.04045 | gene expression | 0.03418 | ligands | 0.02634 | protein folding | 0.02408 |
| Software | 0.01500 | Genome | 0.01428 | models, molecular | 0.01130 | hydrogen bonding | 0.01686 |
| Databases | 0.01365 | Genomics | 0.01238 | structure-activity relationship | 0.00975 | membrane proteins | 0.01489 |
| models, theoretical | 0.01255 | Genotype | 0.01200 | pharmaceutical preparations | 0.00911 | hydrophobicity | 0.01318 |
| cluster analysis | 0.01171 | genes, vpr | 0.00904 | molecular structure | 0.00793 | protein structure, tertiary | 0.00938 |
| Dougherty Edward R | 0.00024 | Churchill Gary A | 0.00013 | Guseinov Israfil | 0.00017 | Baker David | 0.00040 |
| Noble William Stafford | 0.00024 | Pomp Daniel | 0.00012 | Höltje Hans-Dieter | 0.00012 | Dobson Christopher M | 0.00025 |
| Zhao Hongyu | 0.00019 | Womack James E | 0.00011 | Mancera Ricardo L | 0.00009 | Serrano Luis | 0.00020 |
| Zimmer Ralf | 0.00018 | Smith Timothy P L | 0.00010 | Roy Kunal | 0.00009 | Raleigh Daniel P | 0.00018 |
| Bateman Alex | 0.00016 | Garrett Michael R | 0.00008 | Förner Wolfgang | 0.00008 | Thornton Janet M | 0.00018 |
| Bioinformatics (Oxford, England) | 0.80507 | Genomics | 0.38870 | Biochemistry | 0.29003 | Journal of molecular biology | 0.65192 |
| BMC bioinformatics | 0.19257 | Mammalian genome : official journal of the International Mammalian Genome Society | 0.21556 | Journal of computer-aided molecular design | 0.20116 | Protein science : a publication of the Protein Society | 0.33584 |
| Evolutionary bioinformatics online | 0.00216 | Physiological genomics | 0.20318 | Journal of molecular modeling | 0.15709 | Trends in biochemical sciences | 0.01058 |
| Journal of molecular biology | 0.00006 | Human molecular genetics | 0.08093 | Journal of molecular biology | 0.10302 | PLoS computational biology | 0.00122 |
| Proteomics | 0.00001 | BMC genomics | 0.07512 | Protein science : a publication of the Protein Society | 0.05151 | Bioinformatics (Oxford, England) | 0.00013 |
| Topic 16 | | Topic 18 | | Topic 19 | |  |  |
| Protein structure,  Proteomics | | Biotechnology | | Cell structure,  Biotechnology | |  |  |
| protein s | 0.05161 | recombination, genetic | 0.01172 | hela cells | 0.01067 |  |  |
| protein binding | 0.02935 | gene expression | 0.00740 | epithelial cells | 0.01026 |  |  |
| membrane proteins | 0.01718 | recombinant proteins | 0.00723 | cell adhesion | 0.01002 |  |  |
| membranes | 0.01539 | Biomass | 0.00698 | mitochondria | 0.00978 |  |  |
| protein folding | 0.01127 | Kinetics | 0.00607 | cell movement | 0.00717 |  |  |
| Khosla Chaitan | 0.00013 | Pühler Alfred | 0.00022 | Walter Nils G | 0.00006 |  |  |
| Kamo Naoki | 0.00012 | Fussenegger Martin | 0.00010 | Bevilacqua Philip C | 0.00005 |  |  |
| Udgaonkar Jayant B | 0.00011 | Mattiasson Bo | 0.00010 | Wahle Elmar | 0.00004 |  |  |
| Walsh Christopher T | 0.00011 | Olsson Lisbeth | 0.00009 | Aravind L | 0.00004 |  |  |
| London Erwin | 0.00008 | Tramper J | 0.00007 | Varani Gabriele | 0.00004 |  |  |
| Biochemistry | 0.99901 | Journal of biotechnology | 0.64155 | The EMBO journal | 0.21056 |  |  |
| Journal of biotechnology | 0.00021 | Trends in biotechnology | 0.26288 | Biochemistry | 0.18965 |  |  |
| BMC bioinformatics | 0.00015 | Proteomics | 0.03222 | Journal of molecular biology | 0.13980 |  |  |
| Physiological genomics | 0.00015 | Physiological genomics | 0.01833 | Human molecular genetics | 0.07905 |  |  |
| Genomics | 0.00008 | Journal of molecular biology | 0.00837 | Proteomics | 0.05683 |  |  |

**Appendix 3. ACT Model results in third period**

| Topic 0 | | Topic 3 | | Topic 5 | | Topic 6 | |
| --- | --- | --- | --- | --- | --- | --- | --- |
| Hydrogen Bonding,  Molecular Biology | | Proteomics | | Chromosomes,  Genomics | | Gene Expression,  Genomics | |
| oxidoreductases | 0.02408 | gtp-binding proteins | 0.15301 | y chromosome | 0.05103 | gene expression | 0.08948 |
| Oxidants | 0.02324 | proteins | 0.11843 | chromosomes, human, 13-15 | 0.04397 | rna, messenger | 0.02278 |
| hydrogen bonding | 0.02044 | protein s | 0.10385 | chromosomes, human, 1-3 | 0.03739 | gene expression profiling | 0.02191 |
| Oxides | 0.02034 | viral fusion proteins | 0.07356 | chromosomes, human, 19-20 | 0.03100 | promoter regions (genetics) | 0.01999 |
| oxygenators | 0.00891 | protein c | 0.05731 | genome | 0.02661 | up-regulation | 0.01900 |
| Cook Paul F | 0.00027 | Komatsu Setsuko | 0.00020 | Churchill Gary A | 0.00012 | Liu Lin | 0.00007 |
| Gadda Giovanni | 0.00022 | Uhlén Mathias | 0.00013 | Sankoff David | 0.00007 | Yang Jack Y | 0.00007 |
| Fitzpatrick Paul F | 0.00020 | Uversky Vladimir N | 0.00013 | Feil Robert | 0.00007 | Loor Juan J | 0.00006 |
| Bollinger J Martin | 0.00014 | Chou Kuo-Chen | 0.00010 | Lupski James R | 0.00007 | Cheng Chao | 0.00006 |
| Frey Perry A | 0.00012 | Chen Sixue | 0.00010 | Nadeau Joseph H | 0.00006 | Deng Youping | 0.00005 |
| Biochemistry | 0.80218 | Proteomics | 0.34974 | PLoS genetics | 0.20971 | BMC genomics | 0.57542 |
| Journal of molecular biology | 0.13692 | Journal of proteome research | 0.28289 | BMC genomics | 0.09717 | Physiological genomics | 0.10432 |
| Protein science : a publication of the Protein Society | 0.03809 | Molecular & cellular proteomics : MCP | 0.10942 | Human molecular genetics | 0.09497 | Genomics | 0.10017 |
| Trends in biochemical sciences | 0.00446 | BMC genomics | 0.06497 | Mammalian genome : official journal of the International Mammalian Genome Society | 0.06754 | BMC research notes | 0.04994 |
| The EMBO journal | 0.00371 | Journal of biotechnology | 0.04774 | PLoS biology | 0.06099 | Mammalian genome : official journal of the International Mammalian Genome Society | 0.04200 |
| Topic 7 | | Topic 10 | | Topic 11 | | Topic 12 | |
| Protein,  Proteomics | | Analytics,  Proteomics | | Protein,  Proteomics | | Evolution of DNA,  Genetics | |
| gtp-binding proteins | 0.14474 | proteome | 0.13636 | actins | 0.03128 | binding sites | 0.06221 |
| protein s | 0.09534 | proteomics | 0.13207 | tyrosine | 0.01969 | promoter regions (genetics) | 0.03973 |
| gtp-binding protein alpha subunits, gi-go | 0.06737 | spectrum analysis, mass | 0.03305 | ubiquitin c | 0.01745 | dna | 0.03425 |
| protein folding | 0.02264 | spectrometry, mass, matrix-assisted laser desorption-ionization | 0.01633 | ubiquitins | 0.01739 | dna (cytosine-5-)-methyltransferase | 0.03180 |
| binding sites | 0.01411 | signal transduction | 0.01528 | ubiquitin | 0.01510 | down-regulation | 0.02079 |
| Raleigh Daniel P | 0.00028 | Mann Matthias | 0.00040 | Fushman David | 0.00005 | Yokoyama Shigeyuki | 0.00018 |
| Daggett Valerie | 0.00019 | Aebersold Ruedi | 0.00034 | Yokoyama Shigeyuki | 0.00005 | Severinov Konstantin | 0.00012 |
| Baker David | 0.00019 | Smith Richard D | 0.00030 | Wilmanns Matthias | 0.00004 | Geacintov Nicholas E | 0.00011 |
| Sali Andrej | 0.00019 | Heck Albert J R | 0.00026 | Svergun Dmitri I | 0.00003 | Reich Norbert O | 0.00009 |
| Udgaonkar Jayant B | 0.00017 | Thongboonkerd Visith | 0.00025 | Searle Mark S | 0.00003 | Broyde Suse | 0.00009 |
| Journal of molecular biology | 0.42810 | Journal of proteome research | 0.44620 | Journal of molecular biology | 0.24327 | Journal of molecular biology | 0.53144 |
| Biochemistry | 0.34515 | Proteomics | 0.33181 | Biochemistry | 0.19106 | Biochemistry | 0.41458 |
| Protein science : a publication of the Protein Society | 0.19956 | Molecular & cellular proteomics : MCP | 0.15272 | The EMBO journal | 0.16611 | Protein science : a publication of the Protein Society | 0.04553 |
| Journal of molecular modeling | 0.01750 | Journal of proteomics | 0.04861 | Human molecular genetics | 0.04706 | The EMBO journal | 0.00726 |
| Trends in biochemical sciences | 0.00944 | Briefings in functional genomics & proteomics | 0.00813 | Protein science : a publication of the Protein Society | 0.03589 | Journal of molecular modeling | 0.00026 |
| Topic 13 | | Topic 14 | | Topic 15 | | Topic 16 | |
| Protein Evolution,  Proteomics | | Gene transcription,  Genetics | | Theoretical biology | | Protein Structure | |
| promoter regions (genetics) | 0.03461 | genome | 0.09672 | models, theoretical | 0.06463 | gtp-binding proteins | 0.07904 |
| gtp-binding proteins | 0.02580 | genomics | 0.08533 | population | 0.01833 | viral fusion proteins | 0.05048 |
| protein s | 0.01588 | dna transposable elements | 0.02585 | periodicity | 0.01027 | gtp-binding protein alpha subunits, gi-go | 0.04832 |
| transcription factors | 0.01566 | evolution | 0.02283 | mathematics | 0.00972 | proto-oncogene proteins c-yes | 0.04453 |
| Proteins | 0.01538 | gene duplication | 0.01334 | probability | 0.00917 | proteome | 0.02009 |
| Robinson Richard | 0.00019 | Claverie Jean-Michel | 0.00009 | Iwasa Yoh | 0.00024 | Flower Darren R | 0.00023 |
| Sedwick Caitlin | 0.00010 | Gladyshev Vadim N | 0.00006 | Nowak Martin A | 0.00022 | Rost Burkhard | 0.00021 |
| Wittinghofer Alfred | 0.00008 | Gross Liza | 0.00006 | Perelson Alan S | 0.00017 | Bourne Philip E | 0.00020 |
| Gasser Susan M | 0.00006 | Salzberg Steven L | 0.00006 | Steel Mike | 0.00016 | Lengauer Thomas | 0.00019 |
| Auble David T | 0.00005 | Li Wen-Hsiung | 0.00005 | Masuda Naoki | 0.00016 | Kohlbacher Oliver | 0.00016 |
| The EMBO journal | 0.55412 | BMC genomics | 0.35173 | Journal of theoretical biology | 0.46911 | Bioinformatics (Oxford, England) | 0.40947 |
| PLoS biology | 0.16619 | BMC bioinformatics | 0.08746 | Bulletin of mathematical biology | 0.15699 | BMC bioinformatics | 0.39875 |
| PLoS genetics | 0.15807 | PLoS genetics | 0.08436 | PLoS computational biology | 0.13342 | PLoS computational biology | 0.07952 |
| Trends in biochemical sciences | 0.10940 | Genome biology | 0.07649 | PLoS biology | 0.09845 | Bioinformation | 0.05361 |
| Human molecular genetics | 0.01054 | Genomics | 0.05688 | Journal of computational neuroscience | 0.08146 | Journal of computer-aided molecular design | 0.02968 |
| Topic 17 | | Topic 18 | | Topic 19 | |  |  |
| Hepatitis | | Peptide,  Proteomics | | Genomics | |  |  |
| hepatitis e | 0.00909 | c-peptide | 0.06225 | gene expression | 0.03333 |  |  |
| hepatitis a | 0.00864 | peptide t | 0.03903 | gtp-binding proteins | 0.02625 |  |  |
| Hepatocytes | 0.00858 | peptides | 0.03796 | genome | 0.02372 |  |  |
| Hepatitis | 0.00845 | peptide phi | 0.02875 | genomics | 0.02140 |  |  |
| hepatitis b | 0.00787 | peptide-n4-(n-acetyl-beta-glucosaminyl) asparagine amidase | 0.02378 | genes, vpr | 0.01933 |  |  |
| Nicholson Jeremy K | 0.00006 | Shai Yechiel | 0.00013 | Petsko Gregory A | 0.00034 |  |  |
| Chen Wei Ning | 0.00004 | Epand Richard M | 0.00012 | Ruppin Eytan | 0.00020 |  |  |
| Wang Yulan | 0.00003 | Dobson Christopher M | 0.00012 | Palsson Bernhard Ø | 0.00020 |  |  |
| Holmes Elaine | 0.00003 | Olivera Baldomero M | 0.00010 | Wagner Andreas | 0.00017 |  |  |
| Aronow Bruce J | 0.00003 | Kelly Jeffery W | 0.00009 | Valencia Alfonso | 0.00015 |  |  |
| Physiological genomics | 0.08673 | Biochemistry | 0.57118 | Genome biology | 0.30254 |  |  |
| Biochemistry | 0.08493 | Journal of molecular biology | 0.27688 | PLoS computational biology | 0.16859 |  |  |
| Journal of proteome research | 0.08197 | Protein science : a publication of the Protein Society | 0.08425 | BMC systems biology | 0.14190 |  |  |
| BMC genomics | 0.07669 | The EMBO journal | 0.01522 | Molecular systems biology | 0.11646 |  |  |
| Proteomics | 0.07154 | PLoS computational biology | 0.00886 | Trends in genetics : TIG | 0.08448 |  |  |

**Appendix 4. ACT Model results in fourth period**

| Topic 0 | | Topic 1 | | Topic 4 | | Topic 6 | |
| --- | --- | --- | --- | --- | --- | --- | --- |
| Genetics | | Theoretical Biology | | Protein,  Proteomics | | System Biology | |
| Mutation | 0.04205 | models, theoretical | 0.04905 | protein s | 0.02526 | algorithms | 0.04918 |
| Alleles | 0.01905 | Population | 0.01458 | viral fusion proteins | 0.02082 | models, theoretical | 0.02601 |
| promoter regions (genetics) | 0.01635 | Statistics | 0.01142 | proteins | 0.02077 | statistics | 0.01890 |
| Syndrome | 0.01571 | Algorithms | 0.01035 | gtp-binding protein alpha subunits, gi-go | 0.01937 | computational biology | 0.01390 |
| down syndrome | 0.01536 | Environment | 0.00949 | protein c | 0.01907 | software | 0.01343 |
| Swaroop Anand | 0.00007 | Paninski Liam | 0.00013 | Nielsen Jens | 0.00013 | Stadler Peter F | 0.00014 |
| Eng Charis | 0.00007 | Diesmann Markus | 0.00010 | Bork Peer | 0.00010 | Jiang Tao | 0.00014 |
| Stone Edwin M | 0.00007 | Rotter Stefan | 0.00009 | Sansom Mark S P | 0.00008 | Dougherty Edward R | 0.00014 |
| Jin Peng | 0.00006 | Ascoli Giorgio A | 0.00007 | Wang Jin | 0.00008 | Stoye Jens | 0.00011 |
| Davies Kay E | 0.00006 | Brette Romain | 0.00007 | Troyanskaya Olga G | 0.00007 | Zhao Hongyu | 0.00010 |
| Human molecular genetics | 0.72584 | PLoS computational biology | 0.40481 | PLoS computational biology | 0.40234 | Algorithms for molecular biology : AMB | 0.02860 |
| PLoS genetics | 0.17208 | Journal of computational neuroscience | 0.14892 | BMC systems biology | 0.26408 | BioData mining | 0.01619 |
| Physiological genomics | 0.02049 | PLoS biology | 0.12682 | Journal of theoretical biology | 0.15818 | Source code for biology and medicine | 0.01007 |
| Pharmacogenetics and genomics | 0.01420 | Neuroinformatics | 0.05988 | Molecular systems biology | 0.10661 | EURASIP journal on bioinformatics &systems biology | 0.00535 |
| Mammalian genome : official journal of the International Mammalian Genome Society | 0.01321 | BMC systems biology | 0.04064 | Bulletin of mathematical biology | 0.02138 | Journal of biomedical semantics | 0.00242 |
| Topic 8 | | Topic 9 | | Topic 10 | | Topic 11 | |
| Mechanism,  Molecular biology | | Proteomics | | System Biology | | Genomics | |
| hydrogen bonding | 0.01813 | Proteome | 0.07630 | computational biology | 0.04256 | genome | 0.04582 |
| binding sites | 0.01539 | Proteomics | 0.07400 | database [publication type] | 0.02867 | genomics | 0.04312 |
| Ligands | 0.01451 | gtp-binding proteins | 0.03860 | health resources | 0.01525 | gene expression | 0.02731 |
| gtp-binding proteins | 0.01162 | c-peptide | 0.03786 | automation | 0.01516 | mutation | 0.02062 |
| Electrons | 0.00945 | Proteins | 0.02843 | software | 0.01463 | alleles | 0.01914 |
| Esrafili Mehdi D | 0.00023 | Mann Matthias | 0.00043 | Ananiadou Sophia | 0.00014 | Adams David J | 0.00015 |
| Peyghan Ali Ahmadi | 0.00016 | Aebersold Ruedi | 0.00032 | Valencia Alfonso | 0.00013 | Weigel Detlef | 0.00009 |
| Gong Xuedong | 0.00014 | Smith Richard D | 0.00027 | He Yongqun | 0.00013 | Ponting Chris P | 0.00009 |
| Li Yan | 0.00012 | Heck Albert J R | 0.00026 | Cheng Jianlin | 0.00012 | Kruglyak Leonid | 0.00009 |
| Cysewski Piotr | 0.00012 | Martens Lennart | 0.00021 | Kihara Daisuke | 0.00011 | Lehner Ben | 0.00008 |
| Journal of molecular modeling | 0.76381 | Journal of proteome research | 0.40442 | Bioinformatics (Oxford, England) | 0.49531 | PLoS genetics | 0.54455 |
| Journal of computer-aided molecular design | 0.15380 | Molecular & cellular proteomics : MCP | 0.28174 | BMC bioinformatics | 0.39138 | Genome biology | 0.22962 |
| Biochemistry | 0.02746 | Proteomics | 0.18466 | Journal of biomedical semantics | 0.05248 | Trends in genetics : TIG | 0.08217 |
| Journal of biotechnology | 0.00826 | Journal of proteomics | 0.12265 | BioData mining | 0.01179 | Genome medicine | 0.07007 |
| Bioinformatics (Oxford, England) | 0.00722 | Molecular systems biology | 0.00399 | Source code for biology and medicine | 0.00959 | Mammalian genome : official journal of the International Mammalian Genome Society | 0.03858 |
| Topic 12 | | Topic 13 | | Topic 15 | | Topic 16 | |
| Gene transcription,  Genetics | | Proteomics | | Cell Biology | | Theoretical Biology | |
| promoter regions (genetics) | 0.03232 | Proteome | 0.12667 | enteroendocrine cells | 0.09113 | models, theoretical | 0.07544 |
| up-regulation | 0.02618 | Proteomics | 0.12493 | cells | 0.07384 | population | 0.02440 |
| down-regulation | 0.02557 | gtp-binding proteins | 0.06572 | cos cells | 0.03769 | mathematics | 0.01212 |
| transcription factors | 0.01470 | protein s | 0.03962 | l cells (cell line) | 0.02775 | periodicity | 0.01181 |
| signal transduction | 0.01262 | viral fusion proteins | 0.02845 | cell differentiation | 0.01279 | probability | 0.01151 |
| Robinson Richard | 0.00016 | Komatsu Setsuko | 0.00029 | Friedman Avner | 0.00007 | Nowak Martin A | 0.00021 |
| Morimoto Richard I | 0.00007 | Righetti Pier Giorgio | 0.00022 | Tyson John J | 0.00006 | Iwasa Yoh | 0.00020 |
| Tollervey David | 0.00007 | Haynes Paul A | 0.00018 | Lee Peter P | 0.00005 | Steel Mike | 0.00017 |
| Sedwick Caitlin | 0.00006 | Hecker Michael | 0.00014 | Baker Ruth E | 0.00005 | Dieckmann Ulf | 0.00012 |
| Gasser Susan M | 0.00006 | Lubec Gert | 0.00013 | Lindahl Paul A | 0.00005 | Traulsen Arne | 0.00011 |
| The EMBO journal | 0.35979 | Journal of proteomics | 0.38092 | PLoS biology | 0.05751 | Journal of theoretical biology | 0.55404 |
| PLoS genetics | 0.25629 | Proteomics | 0.31010 | Human molecular genetics | 0.03680 | Bulletin of mathematical biology | 0.16476 |
| Journal of molecular biology | 0.14785 | Journal of proteome research | 0.23648 | Genome biology | 0.02119 | Statistical methods in medical research | 0.15705 |
| PLoS biology | 0.12649 | Molecular & cellular proteomics : MCP | 0.06802 | Molecular systems biology | 0.01718 | Theoretical biology &medical modelling | 0.03475 |
| Trends in biochemical sciences | 0.10146 | Physiological genomics | 0.00338 | Molecular & cellular proteomics : MCP | 0.01687 | Trends in biotechnology | 0.03008 |
| Topic 17 | | Topic 18 | | Topic 19 | |  |  |
| Protein Binding | | Evolution of DNA | | Genomics | |  |  |
| gtp-binding proteins | 0.06918 | binding sites | 0.04977 | genome | 0.01618 |  |  |
| protein s | 0.04720 | Kinetics | 0.02047 | binding sites | 0.01453 |  |  |
| gtp-binding protein alpha subunits, gi-go | 0.03247 | Mutation | 0.01525 | genomics | 0.01153 |  |  |
| carrier proteins | 0.01898 | Dna | 0.01218 | gene expression | 0.00894 |  |  |
| protein binding | 0.01555 | hydrogen bonding | 0.01077 | dna | 0.00832 |  |  |
| Udgaonkar Jayant B | 0.00026 | Almo Steven C | 0.00020 | Kumar Anil | 0.00012 |  |  |
| Baker David | 0.00015 | Blanchard John S | 0.00014 | Shapshak Paul | 0.00010 |  |  |
| Raleigh Daniel P | 0.00012 | Holden Hazel M | 0.00014 | Rai Anil | 0.00008 |  |  |
| Sanders Charles R | 0.00012 | Poulos Thomas L | 0.00010 | Velmurugan Devadasan | 0.00008 |  |  |
| Plückthun Andreas | 0.00011 | Walsh Christopher T | 0.00010 | Wadhwa Gulshan | 0.00008 |  |  |
| Biochemistry | 0.47399 | Biochemistry | 0.79877 | BMC genomics | 0.43522 |  |  |
| Journal of molecular biology | 0.32735 | Journal of molecular biology | 0.12732 | Genome biology | 0.06403 |  |  |
| Protein science : a publication of the Protein Society | 0.19147 | Protein science : a publication of the Protein Society | 0.06391 | Genomics | 0.04828 |  |  |
| Journal of computer-aided molecular design | 0.00370 | Trends in biochemical sciences | 0.00258 | Comparative and functional genomics | 0.00889 |  |  |
| Trends in biochemical sciences | 0.00250 | Journal of biotechnology | 0.00217 | Mammalian genome : official journal of the International Mammalian Genome Society | 0.00380 |  |  |
